# Genetic variants of adiponectin receptor 2 are associated with increased adiponectin levels and decreased triglyceride/VLDL levels in patients with metabolic syndrome

**DOI:** 10.1186/1475-2840-5-11

**Published:** 2006-05-15

**Authors:** Uli C Broedl, Michael Lehrke, Elisabeth Fleischer-Brielmaier, Anne B Tietz, Jutta M Nagel, Burkhard Göke, Peter Lohse, Klaus G Parhofer

**Affiliations:** 1 Department of Internal Medicine II, Klinikum Grosshadern, University of Munich, Marchioninistr. 15, 81377 Munich, Germany; 2 Department of Clinical Chemistry, Klinikum Grosshadern, University of Munich, Marchioninistr. 15, 81377 Munich, Germany

## Abstract

**Background:**

Adiponectin acts as an antidiabetic, antiinflammatory and antiatherogenic adipokine. These effects are assumed to be mediated by the recently discovered adiponectin receptors AdipoR1 and AdipoR2.

**Aim:**

The purpose of this study was to determine whether variations in the AdipoR1 and AdipoR2 genes may contribute to insulin resistance, dyslipidemia and inflammation.

**Methods:**

We sequenced all seven coding exons of both genes in 20 unrelated German subjects with metabolic syndrome and tested genetic variants for association with glucose, lipid and inflammatory parameters.

**Results:**

We identified three AdipoR2 variants (+795G/A, +870C/A and +963C/T) in perfect linkage disequilibrium (r^2 ^= 1) with a minor allele frequency of 0.125. This haplotype was associated with higher plasma adiponectin levels and decreased fasting triglyceride, VLDL-triglyceride and VLDL-cholesterol levels. No association, however, was observed between the AdipoR2 SNP cluster and glucose metabolism.

**Conclusion:**

To our knowledge, this is the first study to identify an association between genetic variants of the adiponectin receptor genes and plasma adiponectin levels. Furthermore, our data suggest that AdipoR2 may play an important role in triglyceride/VLDL metabolism.

## Background

Adiponectin (also known as AdipoQ, APM1, and Acrp30) is a hormone secreted by adipocytes that acts as an antidiabetic [1-5], antiinflammatory [6,7], and antiatherogenic [8-10] adipokine. Plasma adiponectin levels are significantly reduced in obesity, insulin resistance, metabolic syndrome, type 2 diabetes, and coronary heart disease [11-17]. Plasma adiponectin concentrations are inversely correlated with HOMA score, insulin levels, visceral and total adipose tissue mass, plasma triglycerides, total and LDL-cholesterol levels [18]. Conversely, adiponectin is directly correlated with VLDL apoB catabolism and HDL-cholesterol levels [18]. Molecularly, adiponectin was shown to increase insulin sensitivity by enhancing insulin's suppressive effect on glucogenesis [2,3] and by increasing fatty acid oxidation in liver and skeletal muscle through activation of AMP kinase and peroxisome proliferator activated receptor (PPAR)-α [1,5,6]. Adiponectin's antiinflammatory and antiatherogenic effects are thought to be mediated by inhibition of TNF-α induced nuclear factor-κB activation [19], inhibition of expression of adhesion molecules [20], decreased smooth muscle cell proliferation [8], and reduced foam cell formation [21].

Recently, two related but distinct receptors for adiponectin were identified, termed adiponectin receptor 1 (AdipoR1) and adiponectin receptor 2 (AdipoR2) [22]. AdipoR1 was shown to be ubiquitously expressed, whereas AdipoR2 expression is more restricted to skeletal muscle and liver. *In vitro *overexpression and knockout experiments demonstrated the receptors' ability to ligand-dependently activate AMP kinase and PPAR-α, and to stimulate fatty acid oxidation and glucose uptake in murine hepatocytes and C2C12 myocytes [22]. Civitarese et al. [23] reported that non-diabetic Mexican- American subjects with a family history of type 2 diabetes exhibited significantly lower levels of AdipoR1 and AdipoR2 mRNA in skeletal muscle compared with those without a family history. Expression levels of both receptors were positively correlated with insulin sensitivity [23].

Assuming that adiponectin receptors AdipoR1 and AdipoR2 mediate the antidiabetic and antiinflammatory effects of adiponectin, we hypothesized that genetic variation in AdipoR1 and AdipoR2 may contribute to insulin resistance, dyslipidemia, and inflammation. We addressed this question by screening AdipoR1 and AdipoR2 for genetic variants and testing for an association between the observed sequence variation and glucose, lipid, and inflammatory parameters in overweight, non-diabetic, insulin resistant subjects.

## Methods

### Ascertainment of subjects

Twenty insulin resistant, abdominal overweight, unrelated German individuals were previously recruited for a randomised, placebo-controlled, double-blind crossover study to determine the effect of telmisartan, an angiontensin type-1 receptor blocker with PPAR-γ activating properties, on glucose and lipid metabolism as well as inflammatory parameters [24]. Insulin resistance was defined as HOMA index ≥ 2.3. Subjects with manifest diabetes mellitus or secondary reasons for insulin resistance (e.g. steroid therapy) were excluded. Abdominal overweight was defined as BMI ≥ 25 kg/m^2^, and waist circumference of ≥ 95 cm in men or ≥ 80 cm in women, respectively. Only subjects with a minimum blood pressure of 120/80 mmHg were included. Subjects with a known history of atherosclerosis (cerebrovascular, peripheral arterial, or coronary) as well as subjects with severe hyperlipoproteinemia (defined as triglycerides >800 mg/dl or LDL-cholesterol >190 mg/dl) were excluded. Other exclusion criteria were a blood pressure of >160/95 mmHg, habitual alcohol consumption of >30 g/d, antihypertensive medication, statin or lipid lowering therapy, consuming illness, or a contraindication against the use of angiotensin II receptor blockers. Subjects were randomised to first receive placebo or telmisartan (40 mg/d) for 12 weeks, after which they received the other medication (telmisartan or placebo). At screening and at the end of each treatment phase primary and secondary parameters were determined. Participating subjects were asked not to change their dietary habits and physical activity throughout the study. The Ethics Committee of the Ludwig-Maximilians-University Munich approved the study protocol and all subjects gave written informed consent.

### Determination of glucose and lipid metabolism parameters

Glucose metabolism was evaluated by fasting plasma glucose (FPG), an oral glucose tolerance test (OGTT), and an intravenous glucose tolerance test (iv GTT). Fasting values included glucose, insulin, and C-peptide concentrations. The OGTT was performed using 75 g glucose following a 12 h fast and was evaluated concerning the (incremental) area under the curve defined by glucose concentrations determined at 0, 30, 60, 90, and 120 min. To calculate the (incremental) area under the insulin curve during OGTT, insulin was also determined at 0, 30, 60, 90, and 120 min. The intravenous glucose tolerance test was performed using 25 g glucose. Both plasma glucose and insulin were measured at 0, 2, 4, 6, 8, and 10 minutes after glucose bolus application. The (incremental) area under the curve for plasma glucose and for insulin was determined. First phase insulin secretion, second phase insulin secretion, insulin resistance index, and the composite index (Matsuda) were calculated based on the data derived from the OGTT as previously described [25].

Triglyceride and cholesterol concentrations were measured using a commercial kit (Boehringer Mannheim, Mannheim, Germany). Preparative ultracentrifugation was performed to isolate VLDL. Cholesterol and triglyceride concentrations were determined in the supernatant and total cholesterol in the infranatant (containing LDL-cholesterol and HDL-cholesterol). After precipitation of apolipoprotein B-containing lipoproteins, HDL-cholesterol was determined in the infranatant. LDL-cholesterol was calculated by subtraction of HDL-cholesterol from total cholesterol in the infranatant. Additionally, postprandial lipoprotein metabolism was evaluated using a standardized oral fat tolerance test. After fasting for 12 hours all subjects ingested a fatty meal, consisting of 100 ml milk (3.5% fat), 150 ml cream (30% fat), 70 ml corn oil, 90 g egg, 10 g sugar and 3.5 g coffee flavour. This standard meal yields 1305 kilocalories, 87% from fat, 7% from carbohydrates, and 6% from protein. Following the fat load, samples were taken every 2 h for 10 h. Total triglyerides and triglycerides in the d < 1.006 g/ml fraction (containing chylomicrons, chylomicron remnants and VLDL) were determined.

To elucidate possible effects on inflammatory processes, high-sensitive (hs-) C-reactive protein (CRP) (Dade Behring), interleukin-6 (IL-6) (R&D Systems), fibrinogen (Dade Behring), and adiponectin (R&D Systems) concentrations were determined.

### Identification of adiponectin receptor variants

Genotyping was performed on above described 20 unrelated subjects. Polymerase chain reaction (PCR) primers for the seven coding exons of both adiponectin receptors were selected in intronic sequence at least 50 base pairs away from the intron/exon boundaries using VectorNTI (Invitrogen). Genomic DNA was isolated from peripheral blood leukocytes using the QIAamp DNA Blood Midi Kit (Qiagen). PCR reactions containing 100 ng of DNA template were amplified in a final volume of 25 μL by denaturation at 95°C for 5 minutes, followed by 35 cycles (95°C for 45 seconds, 55°C for 45 seconds, and 72°C for 1 minute), and an extension at 72°C for 2 minutes. PCR products were run on a 2% agarose gel, and, after confirmation of a single band with the expected size, were purified using the QIAquick PCR Purification Kit (Qiagen). Purified fragments then underwent DNA cycle sequencing using ABI PRISM Big Dye terminator chemistry (Applied Biosystems) and appropriate sequencing primers. Sequences were aligned using VectorNTI (Invitrogen) software, and electropherograms were viewed using Chromas Version 1.45 program. Allelic variations noted from multiple alignments were verified by inspecting the respective electropherograms.

### Statistical analysis

Association of genetic variants with metabolic parameters was tested using the non-parametric Mann-Whitney test. Results of the metabolic studies are depicted as median and interquartile range.

In silico searches were done at the web site of the National Center for Biotechnology Information's database SNP (dbSNP) [26] to determine if any polymorphic sites identified through our sequencing matched those already found.

## Results

All seven coding exons of the AdipoR1 and AdipoR2 genes were sequenced in 20 abdominal overweight (BMI 31.0 (29.3–34.6) kg/m^2^, waist to hip ratio 0.88 (0.83–0.93)), insulin resistant (HOMA 3.35 (2.52–4.43)), unrelated German individuals previously recruited for a randomised, placebo-controlled, double-blind crossover study to determine the effect of telmisartan, an angiotensin type-1 receptor blocker with PPAR-γ activating properties, on glucose and lipid metabolism as well as inflammatory parameters [24]. DNA sequence analysis of the AdipoR1 gene revealed one genetic variation not yet reported in dbSNP (Table 1). In the AdipoR2 gene, sequencing identified seven variants with two of them previously reported in dbSNP (Table 1). Interestingly, three AdipoR2 variants (+795G/A (rs16928751), +870C/A (Ile290Ile) and +963C/T (rs9805042)) were in perfect linkage disequilibrium (r^2 ^= 1) and showed a minor allele frequency of 0.125. Therefore, we decided to focus on this haplotype to study the impact of genetic variants of AdipoR2 on glucose, lipid, and inflammatory parameters in above described subjects.

**Table 1:** Variations in the seven coding exons of the adiponectin receptor 1 and 2 genes

	**SNP name**	SNP location (bp*)	**Variation (major/minor allele)**	**Minor allele frequency**
**AdipoR1**	Ile321Ile	963	T/C	0.05
				
**AdipoR2**	Thr5Thr	15	A/C	0.025
	Val182Gly	545	GTG(Val)/GGG(Gly)	0.025
	Gln244His	732	CAA(Gln)/CAC(His)	0.025
	rs16928751	795	G/A	*0.125*
	Pro272Pro	816	T/C	0.025
	Ile290Ile	870	C/A	*0.125*
	rs9805042	963	C/T	*0.125*

*cDNA base pair location relative to the ATG start site

**Figure 1:**
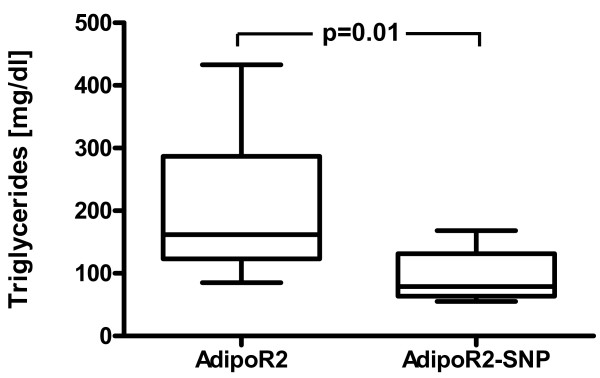
Fasting triglyceride levels [mg/dl] in subjects without (AdipoR2) and with the AdipoR2 SNP cluster (AdipoR2-SNP) after 12 weeks of placebo treatment. Data are presented as box plot (minimum, 25% quartile, median, 75% quartile, maximum).

**Figure 2:**
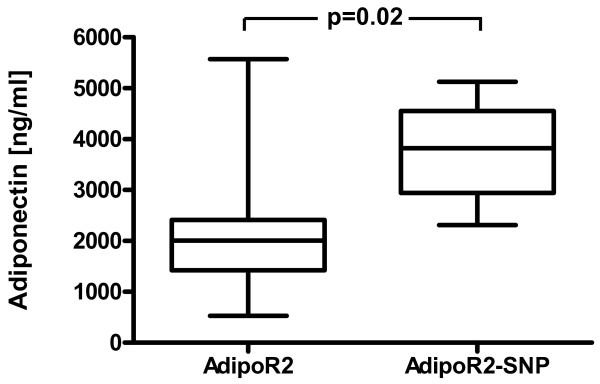
Adiponectin levels [ng/ml] in subjects without (AdipoR2) and with the AdipoR2 SNP cluster (AdipoR2-SNP) after 12 weeks of placebo treatment. Data are presented as box plot (minimum, 25% quartile, median, 75% quartile, maximum).

Subjects with (AdipoR2-SNP) and without the AdipoR2 SNP cluster (AdipoR2) did not differ in age, BMI, waist-to-hip ratio, parameters of glucose metabolism, inflammatory parameters, total cholesterol, HDL- and LDL-cholesterol concentrations (Tables 2,3). However, fasting triglycerides were consistently significantly lower in subjects with the AdipoR2 SNP cluster at three different time points (baseline, end of placebo treatment (Figure 1), end of telmisartan treatment) (Tables 2,3). Furthermore, the AdipoR2 SNP cluster showed a trend for association with reduced fasting VLDL-triglyceride and fasting VLDL-cholesterol levels (Table 3). No association was found between the AdipoR2 SNP cluster and parameters of postprandial triglyceride metabolism (Table 3). Interestingly, the AdipoR2 cluster was associated with significantly lower adiponectin levels (Table 3, Figure 2) at two different timepoints and showed a trend for association with lower adiponectin levels at one timepoint (Table 2).

**Table 2:** Baseline characteristics of subjects without (AdipoR2) and with the AdipoR2 SNP cluster (AdipoR2-SNP). Data are presented as median and interquartile range.

	**AdipoR2**	**AdipoR2-SNP**	**p**
n (female/male)	15 (10/5)	5 (3/2)	
Age [years]	34 (25–44)	41 (27–53)	0.50
BMI [kg/m^2^]	30.7 (29.4–34.5)	31.3 (27.3–35.7)	0.87
Waist to hip ratio	0.89 (0.84–0.93)	0.84 (0.78–0.93)	0.50
Fasting plasma glucose [mg/dl]	105 (100–107)	103 (91–113)	0.74
HOMA-index	3.26 (2.44–4.55)	3.43 (2.74–4.31)	0.93
Fasting total cholesterol [mg/dl]	208 (176–229)	188 (150–227)	0.67
Fasting HDL-cholesterol [mg/dl]	52 (40–60)	57 (51–65)	0.31
Fasting LDL-cholesterol [mg/dl]	114 (85–147)	122 (71–142)	0.87
Fasting triglycerides [mg/dl]	176 (118–285)	83 (72–156)	**0.01**
Adiponectin [ng/ml]	2026 (1477–2470)	3707 (2459–5194)	**0.07**

HOMA: homeostasis model assessment

**Table 3:** Parameters of glucose metabolism, lipid metabolism, and inflammation in patients without (AdipoR2) and with the adiponectin receptor 2 SNP cluster (AdipoR2-SNP) after 12 weeks of placebo or telmisartan treatment. Data are presented as median and interquartile range.

	**Placebo treatment**			**Telmisartan treatment**		
	**AdipoR2**	**AdipoR2-SNP**	**p**	**AdipoR2**	**AdipoR2-SNP**	**p**
Fasting plasma glucose [mg/dl]	100 (94–105)	103 (100–114)	0.44	99 (94–107)	103 (97–114)	0.53
Fasting insulin [μU/ml] *	11.5 (10.0–20.6)	16.7 (8.1–36.8)	0.55	11.9 (7.6–17.7)	14.0 (7.8–20.0)	0.61
Fasting C-peptide [ng/ml]	2.50 (2.20–2.90)	3.30 (1.90–4.45)	0.55	2.50 (2.20–3.10)	2.70 (1.60–3.35)	0.80
1st phase insulin secretion [pmol/l] *	554 (346–882)	536 (308–814)	1.00	519 (359–698)	565 (452–820)	0.61
2nd phase insulin secretion [pmol/l] *	176 (135–246)	171 (125–235)	0.93	164 (131–207)	185 (151–238)	0.61
Glucose iAUC iv GTT [mg*min/dl]^†^	1753 (1507–1905)	1818 (1555–3497)	0.47	1600 (1163–1866)	1510 (1326–1914)	0.80
Glucose iAUC oral GTT [mg*h/dl] *	90 (59–111)	79 (74–104)	1.00	86 (48–105)	67 (31–106)	0.50
Insulin iAUC iv GTT [μU*min/ml]^†^	388 (278–1137)	633 (227–1119)	0.82	407 (297–591)	827 (359–1004)	0.35
Insulin iAUC oral GTT [μU*h/ml] *	110 (71–198)	74 (64–105)	0.27	128 (60–164)	65 (50–156)	0.44
Insulin sensitivity index *	-0.07 (-0.25–0.02)	-0.04 (-0.26–0.07)	0.50	-0.05 (-0.28–0.07)	0.02 (-0.25–0.05)	1.00
Insulin sensitivity index composite *	2.81 (2.20–4.28)	2.61 (2.10–5.67)	0.93	2.60 (1.84–4.79)	2.62 (2.47–4.66)	0.82
HOMA-index	2.95 (2.56–4.77)	4.28 (2.01–10.0)	0.50	2.87 (1.74–4.40)	3.52 (1.90–5.67)	0.50
HbA1c [%]	5.50 (5.20–5.50)	5.40 (5.25–5.65)	0.93	5.35 (5.30–5.65)	5.30 (5.30–5.70)	0.82
						
Fasting total cholesterol [mg/dl]	189 (160–224)	177 (146–209)	0.61	182 (164–222)	176 (140–204)	0.67
Fasting HDL-cholesterol [mg/dl]	48 (36–58)	52 (43–60)	0.61	43 (38–56)	49 (44–60)	0.35
Fasting LDL-cholesterol [mg/dl]	100 (81–137)	114 (76–137)	0.93	96 (73–125)	114 (73–137)	0.61
Fasting TG [mg/dl]	162 (123–287)	79 (64–132)	**0.01**	193 (144–302)	105 (79–113)	**<0.01**
Fasting VLDL-TG [mg/dl]	108 (56–208)	51 (34–91)	**0.07**	126 (68–228)	59 (38–79)	**0.03**
Fasting VLDL-cholesterol [mg/dl]	29 (18–42)	8 (8–25)	**0.06**	32 (15–49)	11 (8–16)	**0.03**
TG iAUC fat tolerance [mg*h/dl] ‡	772 (342–1501)	519 (383–1294)	0.75	931 (438–1380)	632 (343–1142)	0.50
VLDL-TG iAUC fat tolerance [mg*h/dl] ‡	293 (236–611)	238 (87–637)	0.44	317 (149–580)	371 (199–376)	0.75
Chyl-TG iAUC fat tolerance [mg*h/dl] ‡	529 (301–943)	402 (313–756)	0.96	639 (313–1164)	589 (184–764)	0.39
						
Adiponectin [ng/ml]	2008 (1425–2413)	3822 (2944–4553)	**0.02**	2014 (1465–2574)	4573 (2912–4787)	**0.04**
High sensitivity CRP [mg/l]	6.23 (2.26–8.06)	2.50 (1.54–4.19)	0.10	6.12 (2.85–9.85)	2.25 (1.38–4.81)	0.07
Interleukin-6 [pg/ml]	1.44 (1.11–2.20)	1.17 (0.77–2.11)	0.55	1.46 (1.24–2.52)	1.35 (1.03–2.39)	0.93
Fibrinogen [mg/dl]	418 (366–479)	410 (330–470)	0.61	415 (375–477)	401 (352–475)	0.67

* refers to oral glucose tolerance test; ^† ^refers to iv glucose tolerance test; ^‡ ^refers to oral fat tolerance test; HOMA: homeostasis model assessment; iAUC: incremental area under the curve; TG: triglycerides

## Discussion

Our study was designed to test the hypothesis that genetic variation in the recently identified adiponectin receptor genes may contribute to insulin resistance, dyslipidemia, and inflammation. By sequencing all seven coding exons of the AdipoR1 and AdipoR2 genes in overweight, non-diabetic, insulin resistant subjects we identified a common AdipoR2 SNP cluster that was associated with 1) increased adiponectin levels and 2) decreased fasting triglyceride, VLDL-triglyceride and VLDL-cholesterol levels. Our study therefore suggests that AdipoR2 may play an important role in triglyceride/VLDL metabolism. Furthermore, to our knowledge, this is the first study to report an association between adiponectin receptor gene variants and plasma adiponectin levels. 

Stefan et al. [27] recently reported the identification of seven variants of AdipoR2 including the three exonic variants +795G/A, +870C/A and +963C/T referred to as the AdipoR2 SNP cluster in the present study. By examining 502 German non-diabetic patients, Stefan et al. [27] showed these variants to be in high linkage disequilibrium (r^2 ^≥ 0.95) with a minor allele frequency of 0.12 consistent with the results of our study. In contrast to our results, however, Stefan et al. [27] did not find an association between these variants and adiponectin or triglyceride levels. This discrepancy may be attributed to different characteristics of the study populations. Subjects in the present study showed a markedly higher BMI (means: 31.8 versus 26.4 kg/m^2^) and insulin resistance index (HOMA (means): 3.78 versus 1.82), lower adiponectin levels (means: 2586 versus 10844 ng/ml) and, considering these differences, most likely higher lipid parameters (Stefan et al. [27] did not present actual lipid data in their manuscript).

Of course, small numbers of subjects can lead to erroneous results and conclusions. However, determination of metabolic parameters at three different timepoints (0, 12 and 24 weeks) yielded consistent and reproducible results, thus making random associations unlikely.

Adiponectin acts as an antidiabetic, antiinflammatory, and antiatherogenic adipokine. Civitarese et al. [23] reported that in non-diabetic Mexican Americans skeletal muscle specific expression levels of AdipoR2 but not AdipoR1 were positively correlated with plasma adiponectin concentrations. Consistent with these data, we demonstrate that genetic variants of AdipoR2 were associated with higher plasma adiponectin levels.

Furthermore, we show that these variants were associated with lower fasting triglyceride, VLDL-triglyceride and VLDL-cholesterol levels suggesting an important role of adiponectin receptor 2 in triglyceride/VLDL metabolism. Staiger et al. [28] demonstrated that AdipoR2 mRNA expression in human myotubes was only associated with plasma triglyceride levels, whereas AdipoR1 mRNA expression was positively correlated with *in vivo *insulin and C-peptide concentrations, first phase insulin secretion, plasma triglyceride and cholesterol concentrations. The lack of association between genetic variants of AdipoR2 and the triglyceride iAUC in our study suggests that AdipoR2 does not play a role in chylomicron secretion or hydrolysis. In contrast, association with fasting triglyceride, VLDL-triglyceride and VLDL-cholesterol concentrations would be consistent with a role of AdipoR2 in the metabolism of endogenous triglyceride-rich lipoproteins and possibly postprandial remnant lipoproteins. Ng et al. [18] previously reported that adiponectin was inversely associated with plasma triglyceride levels and directly correlated with VLDL apoB catabolism.

The underlying mechanism resulting in an association of the AdipoR2 SNP cluster with increased adiponectin levels and decreased triglyceride/VLDL concentrations still needs to be determined. We hypothesize that these genetic variants, all of them being silent mutations, are in linkage disequilibrium with another yet unidentified functional variation in the AdipoR2 gene.

Damcott et al. [29] recently reported that genetic variation in both adiponectin receptor genes was associated with type 2 diabetes. However, all these genetic variants were non-exonic variants and could therefore not be examined in the present study. Our data did not show any association between the AdipoR2 SNP cluster and glucose metabolism in overweight, non-diabetic, insulin resistant patients.

In conclusion, our data provide the first evidence for an association between variation in the AdipoR2 gene and plasma adiponectin concentrations as well as triglyceride and VLDL levels. Larger studies are necessary to confirm the results of this pilot study.

## Abbreviations

adiponectin receptor 1 (AdipoR1)

adiponectin receptor 2 (AdipoR2)

apolipoprotein B (apoB)

body mass index (BMI)

hemoglobin A1c (HbA1c)

high density lipoprotein (HDL)

homeostasis model assessment (HOMA)

incremental area under the curve (iAUC)

intravenous glucose tolerance test (ivGTT)

low density lipoprotein (LDL)

oral glucose tolerance test (OGTT)

peroxisome proliferator activated receptor (PPAR)

single nucleotide polymorphism (SNP)

single nucleotide polymorphism database (dbSNP)

tumor necrosis factor (TNF)

very low density lipoprotein (VLDL)

## Competing interests

UCB, ML, EFB, ABT, JMN, BG and PL declare that they have no competing interests. KGP was supported by a research grant from Bayer-Vital.

## Authors' contributions

UCB and KGP conceived and designed the study and drafted the manuscript. EFB and PL performed the experiments in this study. ABT, JMN and BG recruited the study subjects and participated in the study design and interpretation of the data analysis. All authors read and approved the final manuscript.
